# An NR2B-Dependent Decrease in the Expression of trkB Receptors Precedes the Disappearance of Dopaminergic Cells in Substantia Nigra in a Rat Model of Presymptomatic Parkinson's Disease

**DOI:** 10.1155/2012/129605

**Published:** 2012-06-07

**Authors:** Eduardo Riquelme, Jorge Abarca, Jorge M. Campusano, Gonzalo Bustos

**Affiliations:** ^1^Departamento de Biología Celular y Molecular, Facultad de Ciencias Biológicas, Pontificia Universidad Católica de Chile, Alameda 340, 8331150 Santiago, Chile; ^2^Programa de Biomedicina, Universidad San Sebastián, Zañartu 1482, Ñuñoa, 7780272 Santiago, Chile

## Abstract

Compensatory changes occurring during presymptomatic stages of Parkinson's disease (PD) would explain that the clinical symptoms of the disease appear late, when the degenerative process is quite advanced. Several data support the proposition that brain-derived neurotrophic factor (BDNF) could play a role in these plastic changes. In the present study, we evaluated the expression of the specific BDNF receptor, trkB, in a rat model of presymptomatic PD generated by intrastriatal injection of the neurotoxin 6-OHDA. Immunohistochemical studies revealed a decrease in trkB expression in SN pars compacta (SNc) seven days after 6-OHDA injection. At this time point, no change in the number of tyrosine hydroxylase (TH) immunoreactive (TH-IR) cells is detected, although a decrease is evident 14 days after neurotoxin injection. The decrease in TH-positive cells and trkB expression in SNc was significantly prevented by systemic administration of Ifenprodil, a specific antagonist of NR2B-containing NMDA receptors. Therefore, an NR2B-NMDA receptor-dependent decrease in trkB expression precedes the disappearance of TH-IR cells in SNc in response to 6-OHDA injection. These results support the idea that a functional coupling between NMDA receptors and BDNF/trkB signalling may be important for the maintenance of the dopaminergic phenotype in SNc during presymptomatic stages of PD.

## 1. Introduction

Parkinson's disease (PD) a progressive degenerative disorder that is characterized by the disappearance of dopaminergic neurons of the nigrostriatal pathway. The clinical symptoms of PD develop slowly and gradually and are only evident after 50–60% of dopamine (DA) cells loss in substantia nigra (SN) and 70–80% decrease of striatal DA content has occurred [1–4]. Compensating responses and plastic changes in the dopaminergic nigrostriatalsystem during presymptomatic PD would be responsible for the delay in the appearance of the clinical symptoms of the disease [5–10]. Emerging evidence suggests that changes in the expression of brain-derived neurotrophic factor (BDNF) in SN may be one of the molecular signals associated with responses occurring in basal ganglia during presymptomatic PD [11]. In agreement with this, a number of studies have demonstrated transient increases of BDNF mRNA and protein in SN, early after partial lesions of the nigrostriatalDA pathway in a rat presymptomatic model of PD [11–13]. These changes in the expression of BDNF could play an important role during the compensatory changes at early stages of PD. This is consistent with reports indicating that BDNF increases the survival of DA neurons [14–17] and that an augmentation of BDNF levels in basal ganglia may prevent degeneration of these neurons in a rat model of PD [18]. Conversely, inhibiting endogenous BDNF expression by antisense oligonucleotide infusion causes loss of nigral dopaminergic neurons in SN [19]. Interestingly, the disappearance of dopaminergic neurons in SN has been also observed when BDNF levels are normal, but its ability to bind or activate its specific receptor, tropomyosin-related kinase B (trkB), has been impaired [20, 21]. These findings indicate the importance of trkB receptor activation in order to generate a full BDNF-induced response in SN. Along this idea, old mutant mice showing haploinsufficiency for trkB exhibit a greater loss of DA neurons in the SN when compared to old wild-type animals [17], which further suggests a possible participation of this receptor in the development of PD.

TrkB is a tyrosine kinase-type receptor, which belongs to the family of trk receptors that binds neurotrophins, event linked to cell survival and synaptic plasticity [22–24]. TrkB and BDNF are both expressed in dopaminergic neurons located in SN [25–28], which suggests that BDNF exerts autocrine/paracrine functions in this nucleus.

We have recently reported a coupling between increased glutamate release, NMDA receptor activation, and BDNF expression in the adult SN, which represents an important molecular signal triggered in this brain nucleus in response to the early and partial DA loss that occurs in striatal nerve endings during presymptomatic PD [13]. These functional interactions occurring in SN could account in part for adaptive and plastic responses associated with early PD. Conversely, no data are available on the expression of trkB receptors in SN during presymptomatic stages of PD as well as on the possibility that glutamate receptors could modulate trkB expression over the progression of the disease. In the present study, by using immunohistochemistry and in situ hybridization, we evaluated the expression of trkB in SN at different time points in a rat model of presymptomatic PD and compare it to the expression of the DA cell marker, Tyrosine hydroxylase (TH). In addition to this, we also assessed the possibility that glutamate receptors might modulate the expression of trkB receptors in SN. Preliminary version of this data has been previously reported in poster format [29].

## 2. Materials and Methods

### 2.1. Animals

Rats weighing 260–300 g were obtained from the Animal Service Unit at the Pontificia Universidad Catolica de Chile and were handled according to the regulations stipulated by the Bioethics and Biosafety Committee of the Faculty of Biological Sciences, Pontificia Universidad Católica de Chile, and by The Animal Care and Use Committee of FONDECYT, Chile.

### 2.2. 6-Hydroxydopamine (6-OHDA) Lesions

Lesions were carried out as reported [13]. Briefly, adult male Sprague-Dawley rats were anesthetized with chloral hydrate (400 mg/kg, i.p.) and mounted in a stereotaxic apparatus (Stoelting). Twenty micrograms of 6-OHDA in 4 *μ*L of 0.02% ascorbic acid was injected in the right striatum at a rate of 0.5 *μ*L/min. Coordinates for the injection of the neurotoxin were *A*: 1.2 mm, *L*: 2.8 mm, and *V*: 4.5 mm, with respect to Bregma, according to the atlas of Paxinos and Watson [30]. Sham-operated rats were stereotaxically injected with 4 *μ*L of 0.02% ascorbic acid using the same coordinates. The rats were allowed to recover for four, seven, and fourteen days, prior to conducting the experiments described hereinafter.

### 2.3. Preparation of Brain Tissue for Staining

Procedure has been previously reported [13]. Rats were anesthetized with chloral hydrate (400 mg/kg; i.p.) and transcardially perfused with saline (0.9% NaCl), followed by ice-cold fixative solution (3% paraformaldehyde, 15% picric acid, 0.1% glutaraldehyde, in phosphatebuffered saline solution (PBS) (pH 7.4). Brains were removed from the skull and postfixed for 30 min. Brains were then dehydrated in 25% sucrose solution for 48 hr at 4°C. Afterwards, 20–30 *μ*m thick coronal slices were prepared on a cryostat (CM 1510; Leica, Heidelberg, Germany) at 5.2 to 5.6 mm posterior to Bregma, the brain region where SN is located according to the atlas of Paxinos and Watson [30]. When fluorescence-based double immunohistochemistry and nonisotopic in situ hybridization (ISH) studies were carried out, the procedure used was as described here, except that the fixative solution was ice-cold 4% paraformaldehyde in PBS.

### 2.4. Immunohistochemical (IHC) Studies

Free-floating coronal midbrain slices were treated with 0.5% H_2_O_2_, rinsed several times in 0.1 M PBS, and then incubated in blocking solution (3% normal goat serum, 0.02% sodium azide, 0.2% Triton X-100 in PBS) for 60 min. Then, slices were incubated for 72 hours at 4°C with the primary antibodies of interest in blocking solution. The slices were then washed in PBS and reacted with a biotinylated goat anti-rabbit IgG (Vector Laboratories) in PBS containing 0.4% BSA and 0.1% Triton X-100, two hours at room temperature. Afterwards, the slices were rinsed in 0.1 M PBS and the immunoreactivity (IR) was visualized with a standard avidin-biotin-peroxidase reagent (1 : 250 dilution, ABC Elite Kit; Vector Laboratories) for 90 min at room temperature. IHC labelling was revealed with 0.05% diaminobenzidine (in 0.05% NiCl and 0.01% H_2_O_2_-Tris saline buffer, pH 7.6) and then observed under light microscopy and the number of trkB-immunoreactive (trkB-IR) cells in SN determined by image analysis as described hereinafter. For TrkB staining, slices were incubated with a 1 : 1000 dilution of a polyclonal rabbit anti-trkB (sc-12, Santa Cruz Biotechnology). TrkB-like IR signals were totally blocked when brain tissue coronal slices were incubated with anti-trkB antibody together with an excess amount (100X) of the immunizing peptide (sc-12P, Santa Cruz Biotechnology). In addition, negative controls made by omission of the first antibody did not reveal IR signals. A 1 : 5000 dilution of a polyclonal rabbit anti-TH antibody (Calbiochem) was used for IHC labelling of TH-containing neurons, as reported previously by us [12].

For double immnunofluorescent experiments, free-floating coronal brain slices were rinsed in 0.1 M PBS buffer containing 3% normal goat serum, 0.02% sodium azide, and 0.2% Triton X-100, for 1 hr at room temperature. Then, the slices were incubated for 72 hours at 4°C in presence of a 1 : 1000 dilution of the anti-trkB antibody (Santa Cruz Biotechnology) and a 1 : 2000 dilution of a polyclonal mouse anti-TH antibody (Calbiochem). Thereafter, the slices were incubated for 1 hour at room temperature with a 1 : 200 dilution of secondary CY3-conjugated anti-rabbit antibody (Jackson ImmunoResearch) and a 1 : 50 dilution of Fluorescein- (FITC-) conjugated anti-mouse antibody (Jackson ImmunoResearch). Finally, the slices were mounted and coverslipped for fluorescence microscopic analysis.

### 2.5. Nissl Staining

To perform a Nissl staining, coronal brain slices were sequentially immersed in the following solutions: xylene, ethanol (at 100%, 95%, and 70%), water, 0.5% cresyl violet (30 min), water, and ethanol (at 70%, 90%, and 100%). Finally, the tissue slices were mounted on gelatinized glass slides, dried overnight, and observed under light microscopy.

### 2.6. Nonisotopic In Situ Hybridization (ISH) of TrkB

The procedure used was essentially as reported previously by us, with slight modifications [31].

#### 2.6.1. Labelling of Oligonucleotide Probe

Deoxynucleotide probes (41-mer) synthesized by BIOS-Chile (Santiago, Chile) were used for ISH experiments. TrkB antisense probe 5′- GTG GAG GGG ATC TCA TTA CTT TTG TTT GTA GTA TCC CCG AT-3′ was complementary to nucleotides 1880–1920 of the reported trkB sequence (M-55291). One hundred picomoles of trkB probe were 3′ endlabeled by incubation with 55 units of terminal transferase in 20 *μ*L of tailing buffer, in presence of 9 nmol of dATP and 1 nmol of digoxigenin-labeleled deoxyuridine-triphosphate (DIG-dUTP).

#### 2.6.2. Hybridization Reaction and Immunological Detection

Brain coronal sections were rinsed in PBS and then incubated at 62°C for 30 min in a prehybridization solution containing Denhardt's 1X (Denhardt's 100X composition: 2% Ficoll, 2% polyvinil pyrrolidone, 2% BSA) and SSC 4X (SSC 20x composition: 3 M NaCl, 0.3 M sodium citrate). Thereafter, the tissue slices were incubated in presence of 10 pmol/mL trkB DIG-labeled antisense probes, in a buffer containing 50% formamide, 0.6 M NaCl, 20 mM EDTA, and 0.2% lauryl sarcosine, in Tris-HCl, pH 7.5, for 20 hr at 35°C. After hybridization, the slices were rinsed in SSC and then reacted with an anti-DIG antibody conjugated to alkaline phosphatase (Boehringer-Mannheim Gmbh Biochemica, Germany). Reaction was developed using NBT and BCIP (Gibco, MD) as enzyme substrates. Finally, the slices were mounted on gelatinized glass slides, dried overnight, and coverslipped for light microscopy. As a control, the tissue slices were hybridized as explained previously, in presence of an excess (100X) of unlabeled trkB probe. As a different experimental control, the slices were incubated with a sense oligonucleotide probe labeled with DIG-UTP. These controls generated no positive signals (data not shown).

### 2.7. Analysis of TrkB and TH Expression in SN

After IHC or ISH procedures, brain sections were examined under a light microscope (Nikon Labophot-2) equipped with a video camera (Sony CCD-Iris) connected to a Macintosh computer. Positive cells for trkB or TH immunostaining and trkB-DIG labelled cells in SN were counted using the NIH image//ppc 1.61 program. For each experimental paradigm, the number of positive cells was evaluated in three different areas per slice, and results shown hereinafter correspond to data obtained from at least four different rats, in which at least three different slices per rat were studied. Only coronal slices corresponding to 5.2 to 5.6 mm posterior to Bregma were used [30].

The number of cells with positive labelling was counted in photomicrographs (20x magnification), with sample areas of 0.042 mm^2^ for SNc and 0.065 mm^2^ for SN pars reticulata (SNr). Thus, raw results obtained are estimates of cell density in each condition. Results are reported as percentage of cells in the ipsilateral SN compared to the contralateral side (100%) in each experimental condition.

Immunofluorescence results were examined under a Fluoview 100 confocal microscope and an Olympus microscope (Olympus BX51, USA) equipped with a fluorescent system. Positive cells were counted using the Q-Capture Pro software (Q-Imaging, Canada). The number of positive cells for each of the IF was evaluated in each slice as indicated previously, under a 40x magnifications, with sample areas of 0.020 mm^2^ for each SNc. Results represent the percentage of cells in SN compacta that exhibit colabelling of TH and trkB over the total number of TH IF-positive cells present in this midbrain subregion.

### 2.8. Statistical Analysis

All statistical analysis were performed using Prism 4.01 GraphPad software. Data were analyzed by Kruskal-Wallis nonparametric ANOVA, followed by a *U*-test. Values of *P* < 0.05 were considered statistically significant. All data are reported as means ± S.E.M.

## 3. Results

### 3.1. A Decrease in the Expression of TrkB-IR Cells and TrkB mRNA Precedes the Disappearance of TH-IR Cells in SNc of Rats after Unilateral 6-OHDA Intrastriatal Injections

In our laboratory, we have recently used an animal model proposed to imitate the presymptomatic stages of PD [3, 32]. By using this model, which consists of a unilateral intrastriatal injection of the neurotoxin 6-OHDA, we have shown a progressive reduction in rat striatal DA levels 1 to 7 days after neurotoxin injection [13]. We also showed that in SNc, the IR for TH, the key enzyme in the synthesis of DA and a common marker for dopaminergic neurons, is decreased by 14 days after neurotoxin injection [13].

**Figure 1:**
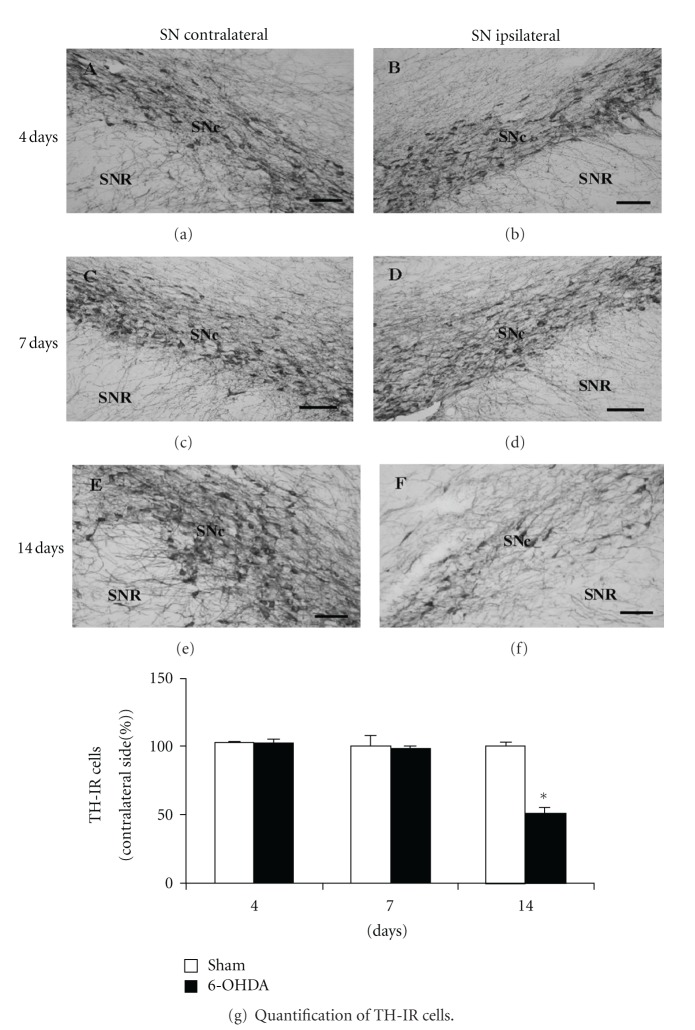
The unilateral intrastriatal injection of 6-OHDA induces a decrease in the number of TH immunoreactive cells in SNc that is only evident 14 days after the neurotoxin injection. Photomicrographs show TH-immunoreactive (IR) cells in the contralateral (a), (c), and (e) and ipsilateral (b), (d), and (f) side of rat SN 4, 7, and 14 days after unilateral 6-OHDA intrastriatal injection, respectively. (a)–(f) scale bar = 100 *μ*m. SNc: substantia nigra pars compacta; SNR: substantia nigra pars reticulata. (g) Percentage of TH-IR cells in the ipsilateral SNc compared to the contralateral side, at 4, 7, and 14 days after unilateral intrastriatal 6-OHDA (black columns) or ascorbic acid (sham, white columns) injections to rats. The number of cells with TH-IR signals per unit area per slice was 507.9 ± 7.8, 484.1 ± 71.4, and 531.7 ± 103.2 cells/mm^2^ in contralateral SNc of ascorbate injected rats, at 4, 7, and 14 days after treatment, respectively. In the case of 6-OHDA-treated rats, the number of cells with TH-IR signals in contralateral SNc was 500.0 ± 23.8, 452.4 ± 87.3, and 507.9 ± 111.1 cells/mm^2^, at 4, 7, and 14 days after neurotoxin injection, respectively. *n* = 6 rats under each experimental condition. The different experimental groups were compared by a Kruskal-Wallis nonparametric ANOVA, followed by a *U*-test. *P < 0.05 compared with sham-treated rats.

Expanding those studies, we analyzed the number of TH-IR cells in SNc of rats 4, 7, and 14 days after unilateral 6-OHDA intrastriatal injection (Figure 1). We did not visualize changes in the number of TH-IR cells in ipsilateral SNc 4 or 7 days after unilateral 6-OHDA intrastriatal injections, when compared to the contralateral SNc of the respective animal (Figures 1(a) and 1(b) and Figures 1(c) and 1(d), at 4 and 7 days, resp.). However, as shown previously [13], we detected a decrease in the number of TH-IR cells in the ipsilateral SNc 14 days after neurotoxin injection compared to its contralateral side (Figures 1(e) and 1(f)). Quantification of data obtained in several animals after 6-OHDA and sham treatment is shown in Figure 1(g). We only detected a statistically significant reduction in the relative number of TH-IR cells in SNc two weeks after striatal neurotoxin administration (48.6% ± 3.4, *P* < 0.01, 6-OHDA versus sham treatment) and no differences were observed at earlier time points (Figure 1(g)). Interestingly, the number of Nissl-stained cells in ipsilateral SNc remained unaffected 4 to 7 days after unilateral 6-OHDA intrastriatal injection (102 ± 4 and 93 ± 5% of cells in ipsilateral SNc compared to contralateral side at 4 and 7 days after neurotoxin injection, resp.; *P* > 0.05). It was only possible to detect a reduction in the number of Nissl-positive cells 14 days after neurotoxin injection (47 ± 7% of cells in ipsilateral SNc compared to the contralateral side; *P* < 0.05). Altogether, these results demonstrate that the number of TH-IR cells and Nissl-stained cells in SNc is only modified 2 weeks after an intrastriatal injection of 6-OHDA, consistent with a progressive model of PD and with previous results from us and others [12, 33–35].

We have previously shown a transient increase in the genic expression of BDNF in the ipsilateral SN as early as 1 day after a unilateral intrastriatal injection of 6-OHDA and prior to the disappearance of TH-IR cells in SNc [13]. In this work, we decided to evaluate the expression of trkB, the specific BDNF receptor, in SN, using the same experimental paradigm.

**Figure 2:**
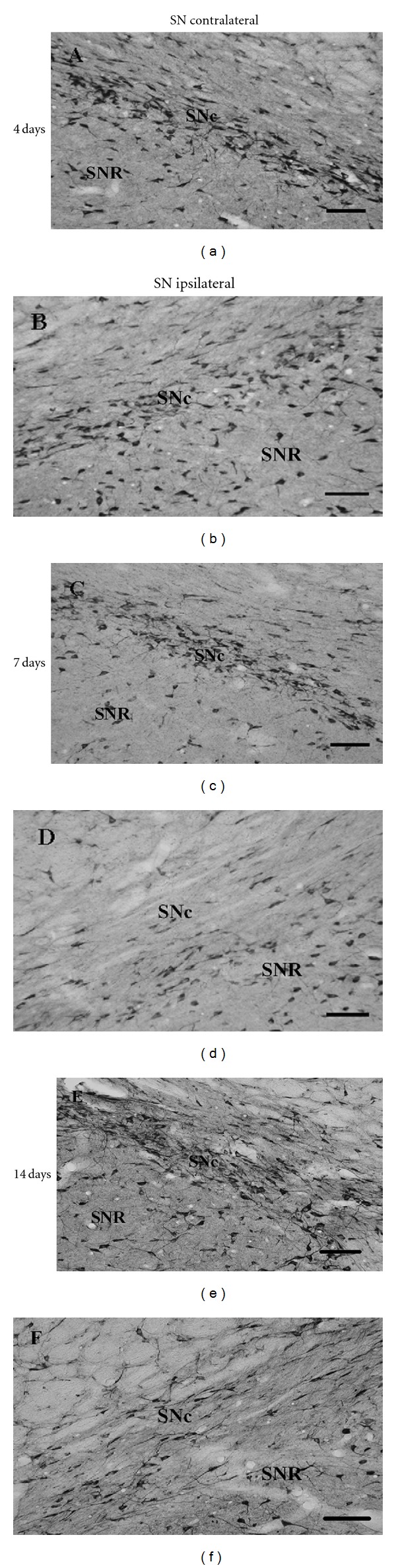
Photomicrographs showing a decrease in the number of trkB-IR cells in ipsilateral SNc at 7 and 14 days, but not at 4 days, after unilateral 6-OHDA intrastriatal injections. Photomicrographs show trkB-immunoreactive (IR) cells in the contralateral (a), (c), and (e) and ipsilateral (b), (d), and (f) sides of rat SN at 4, 7, and 14 days after unilateral intrastriatal injection of 6-OHDA. (a)–(f) scale bar = 100 mm. SNc: substantia nigra pars compacta; SNR: substantia nigra pars reticulata.

**Figure 3:**
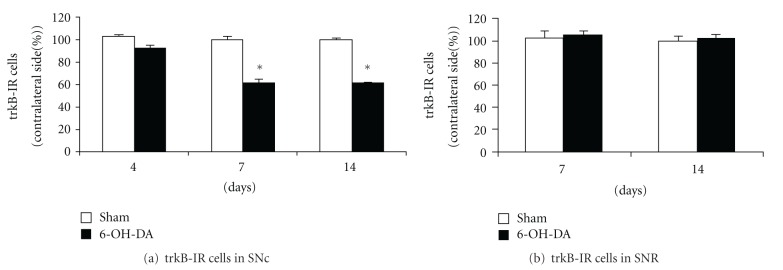
Unilateral 6-OHDA intrastriatal injection results in a significant decrease in the number of trkB-IR cells in ipsilateral SNc at 7 and 14 days after the injection. (a) Percentage of trkB-IR cells in rat ipsilateral SNc compared to the contralateral side after unilateral 6-OHDA (black columns) or ascorbic acid (sham, white columns) intrastriatal injections. Number of cells with trkB-IR signals in contralateral SNc of ascorbate-injected rats per area unit per slice was 507.9 ± 7.9, 365.1 ± 15.9, and 412.7 ± 10.6 cells/mm^2^, at 4, 7, and 14 days after injections, respectively. In the case of 6-OHDA-treated rats, number of cells with trkB-IR signals in contralateral SNc was 484.1 ± 15.9, 381.0 ± 31.7 and 404.7 ± 15.9 cells/mm^2^, at 4, 7, and 14 days after 6-OHDA injections, respectively. (b) Percentage of trkB-IR cells in the ipsilateral SNR compared to the contralateral side, at 7 and 14 days after unilateral intrastriatal injections of 6-OHDA (black columns) or ascorbic acid (sham, white columns). Number of cells with trkB-IR signals in contralateral SNR of ascorbate-treated rats was 117.9 ± 5.1 and 143.6 ± 5.1 cells/mm^2^, at 7 and 14 days after injections, respectively. In the case of 6-OHDA-treated rats, the number of cells with trkB-IR signals in contralateral SNR amounted to 117.9 ± 5.1 and 148.7 ± 10.3 cells/mm^2^, at 7 and 14 days after toxin injections, respectively. *n* = 6 rats under each experimental condition. The different experimental groups were compared by a Kruskal-Wallis nonparametric ANOVA, followed by a *U*-test. *P < 0.05 compared with sham-treated rats.

Photomicrographs shown in Figures 2(a), 2(c), and 2(e), are representative of the distribution of trkB-IR cells in control SN and are consistent with data in the literature [36, 37]. Immunoreactive cells concentrate densely in SNc while it is also possible to detect a small number of labeled cells scattered over the Substantia Nigra pars reticulata (SNR). This pattern of trkB-IR is dramatically affected in the ipsilateral side of SN at 7 and 14 days after unilateral intrastriatal 6-OHDA injection. The disappearance of the dense immunoreactivity for trkB in SNc (Figures 2(d) and 2(f)) is especially remarkable. In contrast, these studies exhibited no change in the distribution of trkB-IR cells in ipsilateral SNc as compared to contralateral SNc 4 days after unilateral 6-OHDA injections (Figures 2(a) and 2(b)). Quantification of the number of trkB-IR cells in ipsilateral SN was performed at 4, 7, and 14 days after 6-OHDA or ascorbic acid (sham-treated) unilateral intrastriatal injections (Figure 3). Data are presented as percent change in the number of trkB-IR cells in ipsilateral SNc (Figure 3(a)) or SNR (Figure 3(b)) compared to the respective contralateral side, in both sham- and 6-OHDA-treated animals. A significant 40 ± 3% and 40 ± 1.5% reduction in the relative number of trkB-IR cells in ipsilateral SNc was detected in 6-OHDA-treated rats 7 and 14 days after intrastriatal injection of the neurotoxin when compared to sham animals, respectively (*P* < 0.05 in each case) (Figure 3(a)). No differences were observed when comparing the relative number of trkB-IR cells in ipsilateral SNc 4 days after unilateral 6-OHDA or ascorbic acid injections (Figure 3(a)). Interestingly, the reduction in trkB-IR cells observed at 7 and 14 days after 6-OHDA seems to be a subregion specific phenomenon in SN since no change in the relative number of trkB-IR cells was detected in ipsilateral SNR at any time point after neurotoxin injection (Figure 3(b)).

**Figure 4:**
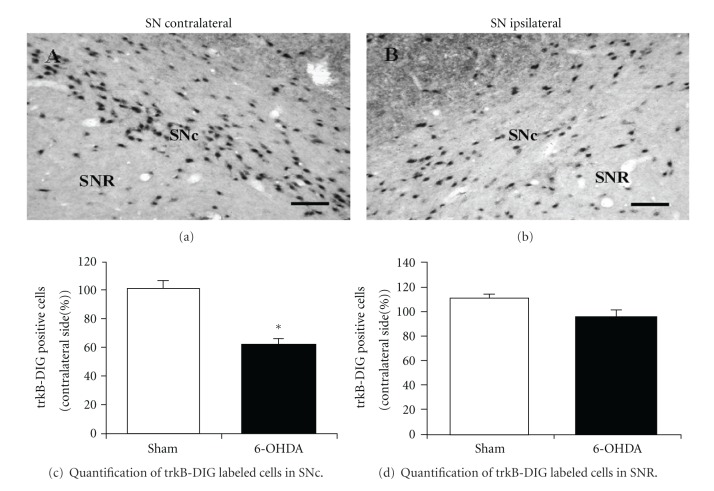
A specific decrease in the number of positive trkB-mRNA cells is observed in ipsilateral SNc 7 days after unilateral 6-OHDA intrastriatal injections. Photomicrographs from ISH experiments show trkB-DIG positive cells in the contralateral side (a) and ipsilateral side (b) of rat SN 7 days after unilateral 6-OHDA intrastriatal injection, respectively. (a), (b) scale bar = 100 *μ*m. SNc: substantia nigra pars compacta; SNR: substantia nigra pars reticulata. (c) Percentage of trkB-DIG positive cells in the ipsilateral SNc compared with the contralateral side, 7 days after unilateral intrastriatal 6-OHDA (black columns) or ascorbic acid (sham, white columns) injections to rats. Number of cells with trkB-DIG-positive signals in contralateral SNc was 285.7 ± 15.9 and 317.5 ± 31.7 cells/mm^2^ in ascorbate and 6-OHDA injected rats, respectively. (d) Percentage of trkB-DIG positive cells in the ipsilateral SNR compared to the contralateral side, 7 days after unilateral intrastriatal 6-OHDA (black columns) or ascorbic acid (sham, white columns) injections to rats. Number of cells with trkB-DIG positive signals in contralateral SNR was 107.7 ± 5.1 and 117.9 ± 5.1 cells/mm^2^ in ascorbate and 6-OHDA injected rats, respectively. *n* = 4 rats under each experimental condition. The different experimental groups were compared with a Kruskal-Wallis nonparametric ANOVA, followed by a *U*-test. *P < 0.05 compared with sham-treated rats.

The aforementioned changes in the number of trkB immunoreactive cells in ipsilateral SNc after 6-OHDA injection could be explained by a transcriptional mechanism; that is, the genic expression of trkB could be reduced in this midbrain nucleus after the neurotoxin striatal injection. To provide evidence regarding this proposition, the distribution and number of cells expressing trkB mRNA in SN were evaluated by nonisotopic ISH seven days after the unilateral 6-OHDA intrastriatal injection. Seven days of 6-OHDA treatment was chosen for these ISH studies as no changes in Nissl-stained cells or in TH-IR cells were detected in ipsilateral SNc at this time point after neurotoxin injection compared to sham rats, as indicated previously. The ISH studies revealed a high concentration of cells expressing trkB mRNA in SNc and a more disperse localization of trkB mRNA-expressing cells in SNR, as it is shown in contralateral SN in Figure 4(a). However, this pattern of trkB mRNA expression is quite different in ipsilateral SN at 7 days after unilateral intrastriatal injection of the neurotoxin (Figure 4(b)), since it is possible to observe a diffuse distribution of DIG-labeled cells throughout the SN in conjunction with a relative decrease in the number of these cells in SNc. The evaluation of the number of trkB-DIG labeled cells in ipsilateral SNc at 7 days after unilateral striatal 6-OHDA injection, expressed as percent of change over the contralateral SNc, revealed a 38 ± 4% decrease compared to sham-treated rats (Figure 4(c), *P* < 0.05). Conversely, no statistical differences were detected when comparing the number of cells expressing the mRNA for trkB in ipsilateral SNR after 6-OHDA or ascorbic acid striatal injections (Figure 4(d)). These results are consistent with those obtained before by IHC and raise the possibility that the specific decrease in the expression of trkB observed in ipsilateral SNc, in response to 6-OHDA injection in the striatum, might be mediated at least in part by a transcriptional mechanism. 

### 3.2. Dopaminergic Neurons of SNc Fail to Express TrkB at Early Stages after 6-OHDA Striatal Administration

The aforementioned results demonstrate that as early as seven days after the unilateral intrastriatal injection of 6-OHDA, it is possible to detect a decrease in the number of trkB-IR cells in SNc (Figures 2 and 3) without a change in the number of TH-positive cells in this midbrain subarea (Figure 1). On the other hand, the total number of cells detected by Nissl staining remained unchanged in SNc at this time point after 6-OHDA treatment. Therefore the early decrease in trkB-IR cells might be the consequence of a reduced expression of trkB in the population of dopaminergic neurons and not due to the disappearance of these neurons in SNc. We decided to evaluate this proposition.

**Figure 5:**
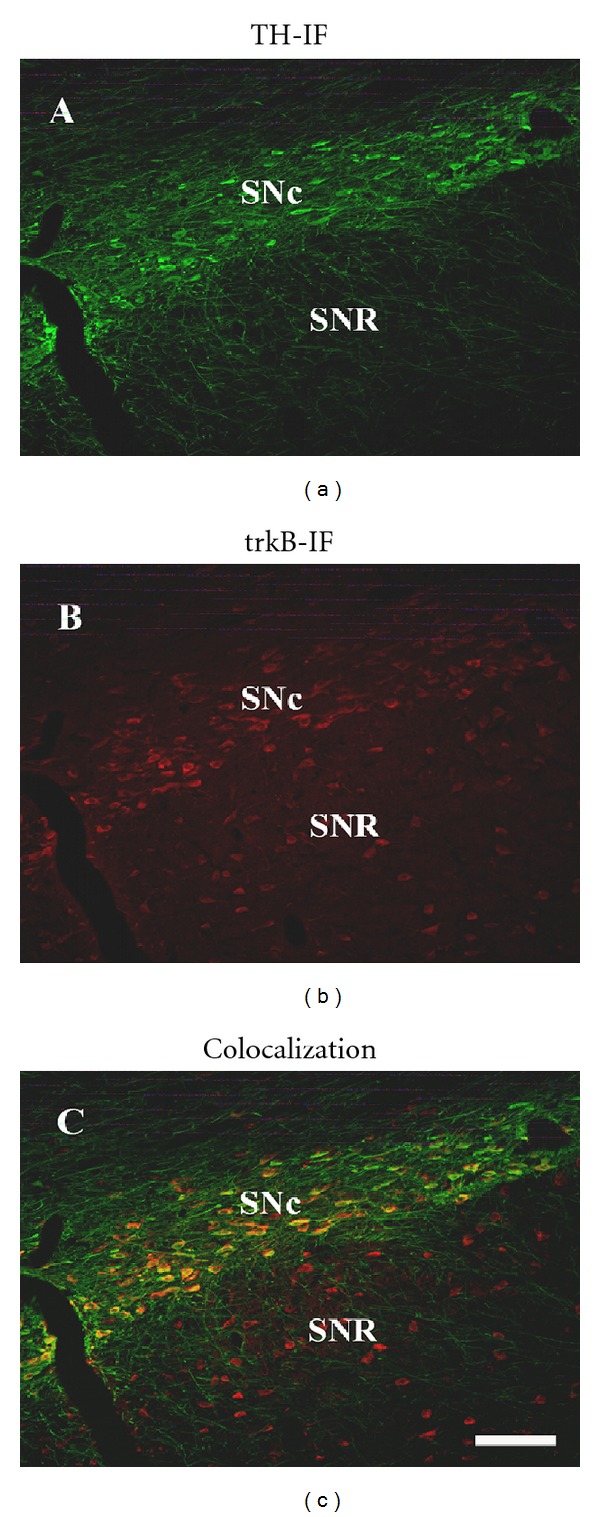
TH and trkB immunoreactivities are highly colocalized in rat SNc. Immunofluorescent detection of TH (a) and trkB (b) in SN in untreated naïve rats. (c) Merge of (a) and (b). (a)–(c) scale bar = 150 *μ*m. SNc: substantia nigra pars compacta; SNR: substantia nigra pars reticulata.

We first studied the expression of trkB in TH-positive cells by double immunofluorescence (IF), in naïve control animals. As expected, TH-IF positive cell bodies are only observed in SNc (Figure 5(a)). Even though there are no TH-positive cell bodies in SNR, it is possible to observe a significant number of TH-IF labeled neurites projecting from the SNc to the SNR (Figure 5(a)). On the other hand, IF studies exhibited trkB-positive cells in both SNc and SNR (Figure 5(b)), in agreement with the IHC results shown before. When both images are merged, it is possible to observe colocalization of TH-IF and trkB-IF in SNc, while this colocalization does not occur in SNR (Figure 5(c)). Indeed, approximately 80% of the total TH-IF cells in SNc were found to coexpress trkB (see the following).

**Figure 6:**
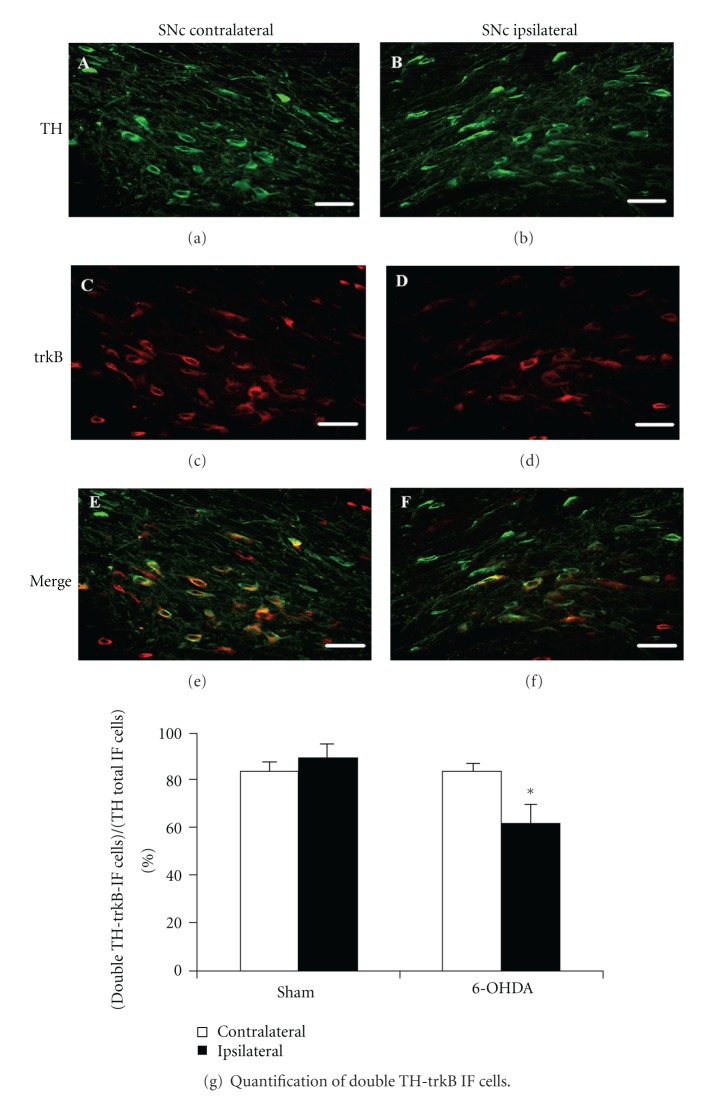
A decrease in the percentage of double labeling for TH and trkB in SNc is detected 7 days after unilateral 6-OHDA intrastriatal injections. Photomicrographs show TH-IF cells (a), (b) and trkB-IF cells (c), (d) in rat SNc, 7 days after unilateral 6-OHDA intrastriatal injection. (e) Merge of (a) and (c); (f) merge of (b) and (d). (a), (c), and (d) correspond to contralateral SNc while (b), (d), and (f) to ipsilateral SNc. (a)–(f) scale bar = 50 *μ*m. SNc: substantia nigra pars compacta. (g) Quantification of cells that coexpress TH and trkB in the ipsilateral SNc compared to their contralateral side, 7 days after unilateral 6-OHDA or ascorbic acid (sham) intrastriatal injections to rats. The results were expressed as the percentage of cells that coexpress TH and trkB over the number of total TH-IF cells. Number of cells with TH-IF signals and double TH-trkB-IF signals in contralateral SNc after ascorbate injection was 365.1 ± 39.7 and 309.5 ± 39.7 cells/mm^2^, respectively. In the case of 6-OHDA-treated rats, number of cells with TH-IF signals and double TH-trkB-IF signals in contralateral SNc was 333.3 ± 23.8 and 277.7 ± 7.9 cells/mm^2^, respectively. *n* = 4 rats for each experimental condition. The different experimental groups were compared by a Kruskal-Wallis nonparametric ANOVA, followed by a *U*-test. *P < 0.05 compared with sham-treated rats.

Immunofluorescence studies conducted 7 days after unilateral intrastriatal injection of 6-OHDA showed results compatible with a reduction in the number of trkB-IF cells in ipsilateral compared to contralateral SNc (Figures 6(c) and 6(d)), while the number of TH-IF cells remained unchanged (Figures 6(a) and 6(b)). In addition, the merge of these pictures indicates a decrease in the number of cells coexpressing TH and trkB in SNc ipsilateral to the striatal injection of 6-OHDA (Figures 6(e) and 6(f)). Indeed, quantification of the results obtained in several animals revealed a statistically significant reduction in the colocalization of TH-IF and trkB-IF cells in ipsilateral SNc compared to its contralateral side after neurotoxin treatment (39 ± 8% reduction, *P* < 0.05) (Figure 6(g)). No such change was observed in sham-treated rats (Figure 6(g)). These results indicate that the early decrease in trkB expression observed in ipsilateral SNc after intrastriatal 6-OHDA injection is most likely due to a reduced expression of the neurotrophin receptor in TH-positive cells occurring in SNc and not to the disappearance of these cells in this midbrain subregion.

### 3.3. Ifenprodil Inhibits Both TrkB Downregulation and TH Decreased Expression in SNc Induced by a Unilateral 6-OHDA Intrastriatal Microinjection

Recent results from us suggest the induction of a coupling between Glutamatergic drive, NMDA receptor activation, and increased BDNF expression in SN at the very early stages of the present rodent model of presymptomatic PD [11, 13]. Therefore, we sought to determine, by means of a Glutamate receptor antagonist, whether NMDA receptors might be involved in the early decrease in trkB expression as well as in the TH-IR cell disappearance that is observed in SNc in response to 6-OHDA intrastriatal injection. The selective NR2B-NMDA receptor antagonist, ifenprodil, was chosen for these studies because of reports indicating that activation of these receptor subtypes might suppress BDNF/trkB receptor complex expression and initiate or facilitate signaling cascades involved in neuronal cell death [38].

**Figure 7:**
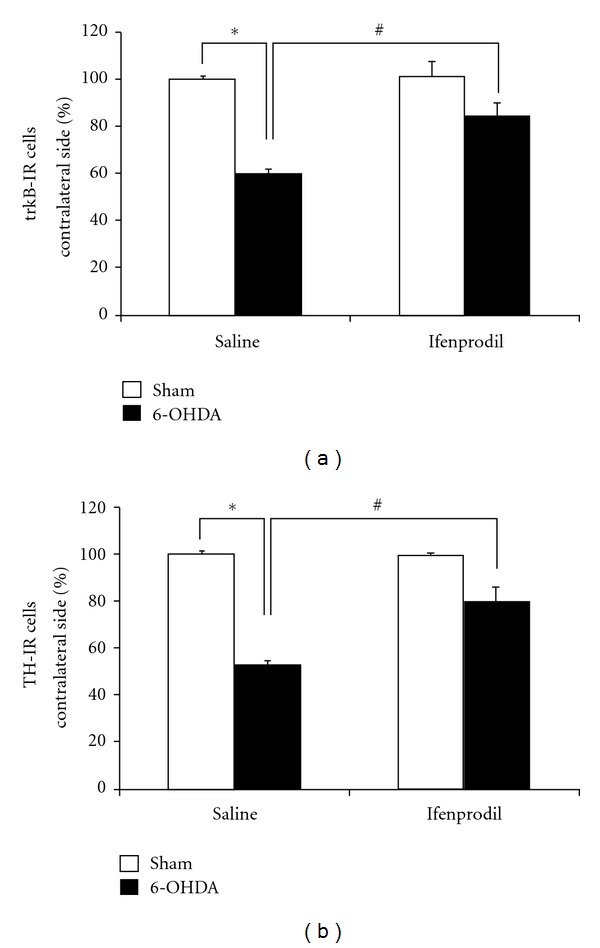
Ifenprodil, an antagonist of NR2B containing NMDA receptors, prevents the decrease in trkB-IR and TH-IR cells occurring in SNc 14 days after 6-OHDA intrastriatal injection. Positive cells for trkB and TH immunostaining in SNc were counted in coronal midbrain slices of treated rats as described in Section 2. Four groups of rats in each case (a) and (b) were pretreated with saline solution or Ifenprodil (5 mg/kg, i.p.), 1 day before and 3, 5, and 7 days after unilateral 6-OHDA (black columns) or ascorbic acid (sham, white columns) intrastriatal administration. The IHC studies were performed 14 days after 6-OHDA or ascorbic acid injections. Results were expressed as a percentage of trkB-IR cells (a) or TH-IR cells (b) in the ipsilateral SNc compared with the contralateral side. (a) Number of cells with trkB-IR signals in sample areas per slice from contralateral SNc was equal to 365.1 ± 15.9, 381.0 ± 31.7, 412.7 ± 10.6, and 404.7 ± 15.9 cells/mm^2^ in sham-saline, 6-OHDA-saline, sham-Ifenprodil, and 6-OHDA-Ifenprodil rats, respectively. (b) Number of TH-IR cells in sample areas per slice from contralateral SNc was equal to 404.8 ± 23.8, 357.1 ± 29.8, 386.9 ± 11.9, and 369.0 ± 6.0 cells/mm^2^ in sham-saline, 6-OHDA-saline, sham-Ifenprodil, and 6-OHDA-Ifenprodil rats, respectively. The different experimental groups were compared by a Kruskal-Wallis nonparametric ANOVA, followed by a *U*-test. *P < 0.05, when comparing saline-6-OHDA versus saline-sham rats. ^#^ *P* < 0.05 when comparing saline-6-OHDA versus Ifenprodil-6OHDA rats. *n* = 3 rats under each experimental condition.

In the following experiments, four groups of rats were treated with consecutive i.p. injections of Ifenprodil (5 mg/kg) or saline solution, administered 1 day before and 3, 5, and 7 days after 6-OHDA or ascorbic acid (sham rats) intrastriatal injection. A marked decrease in the relative expression of trkB-IR cells occurs in the ipsilateral SNc of saline pretreated rats after 14 days of 6-OHDA intrastriatal injection compared with the ipsilateral SNc of sham rats (Figure 7(a), first versus second column, *P* < 0.05). Such 6-OHDA-induced decrease of trkB-IR cells in the ipsilateral SNc was significantly prevented in rats pretreated with Ifenprodil (Figure 7(a), second versus fourth column). Ifenprodil pretreatment also totally prevented the decrease of trkB expression observed in the ipsilateral SNc 7 days after neurotoxin intrastriatal injection (41.4 ± 4% reduction versus 3.5 ± 0.4% reduction in the number of trkB-IR cells in the SNc when comparing 6-OHDA injected rats versus 6-OHDA plus Ifenprodil injected rats, *P* < 0.01). On the other hand, saline and Ifenprodil pretreatment produced no effect on trkB-IR cell numbers, as evidenced in the ipsilateral SNc of sham rats (Figure 7(a), first versus third column).

IHC studies with antibodies against TH were also conducted in Ifenprodil- and saline-treated rats after unilateral 6-OHDA or ascorbic acid (sham) intrastriatal administration. The IHC studies illustrated in Figure 7(b) were performed 14 days after neurotoxin or ascorbate injections. As shown in Figure 7(b), decreases in the number of TH-IR cells in the ipsilateral SNc are readily observed after 6-OHDA striatal injections to saline pretreated rats (columns under saline pretreatment, *P* < 0.05). In contrast, Ifenprodil pretreatment blocked the appearance of such 6-OHDA-induced decrease of TH-IR cells observed in SNc (Figure 7(b)). On the other hand, decreases in the number of TH-IR cells in SNc were not detected 7 days after 6-OHDA injection (Figure 1) and the expression of TH observed at this time point was not statistically modified by ifenprodil pretreatment (*P* > 0.05).

Therefore, these studies show that trkB expression in SNc of rats may be decreased after a partial lesion of the nigrostriatal DAergic neuronal pathway induced by 6-OHDA and that this decrease along with that of TH-IR occurring in SNc may be prevented by NR2B-containing NMDA receptors antagonists such as Ifenprodil.

## 4. Discussion

### 4.1. The 6-OHDA Presymptomatic Rat Model of Parkinson Disease

One of the main pathological hallmarks of PD is the progressive and selective loss of dopaminergic neurons in SN. The clinical symptoms of PD appear when striatal DA is depleted 70–80% and when 50–60% of DA cell loss has occurred in SNc [1, 2, 4, 39]. In the present work, we have used a rat model that closely mimics the neurochemical characteristics of early PD, producing a slow degeneration of the dopaminergic nigrostriatal pathway over a period of several weeks [32, 34, 40, 41]. In essence, this model of presymptomatic PD is produced by an intrastriatal unilateral injection of 6-OHDA, which initially induces a partial damage (20 to 50%) of DA terminals in the striatum followed by a slow progressive degeneration of dopaminergic cells in the ipsilateral SN [13, 32]. In these conditions, we were only able to observe a significant reduction in the number of TH-IR cells in SN starting fourteen days after 6-OHDA injection, in agreement with previous results from us and others [12, 13, 34, 40]. Indeed, this PD rat model allowed us to study changes in the expression of trkB receptors in SN that might occur prior to any reduction in the number of dopaminergic neurons in this midbrain area.

### 4.2. Intrastriatal 6-OHDA Injection Induces Changes in the Expression of TrkB in SNc

Recent studies in our laboratory have shown an increased expression of BDNF transcripts in SN as early as one day after 6-OHDA intrastriatal injection [13]. However, nigral BDNF expression returned to basal levels by 7 days after neurotoxin injection. Therefore, a transient upregulation of BDNF expression in SN seems to exist at early stages in this rodent model of PD. We have proposed that these changes in BDNF transcripts may constitute part of compensatory actions triggered to maintain the survival and integrity of DA cells in SN [11, 13]. In this work we decided to expand our knowledge on this model of presymptomatic PD by evaluating the expression of the BDNF receptor, trkB. We detected a decrease in trkB expression in SNc 7 days after 6-OHDA injection. This reduction in the expression of trkB after 6-OHDA treatment does not seem to be accounted by dopaminergic cell death as no change in the number of TH-positive cells or in the total number of cells was detected in SNc at this time point. Indeed, our studies demonstrate that 83% of TH-positive cells in SNc coexpress trkB, which is in agreement with previous results from others [26]. No colocalization was detected in SNR, as no TH-positive cells were observed in this SN subregion [26, 36, 37, 42]. In addition, by seven days after unilateral intrastriatal injection with the neurotoxin it was possible to detect a reduction in the colocalization of trkB and TH in ipsilateral SNc compared to the control contralateral SNc. Since at this time point we did not detect any change in the number of TH-positive cells or in the total number of cells in this brain region, these results suggest that the decrease in trkB receptor expression after 6-OHDA treatment might be due to a reduction in the genic expression of this receptor in TH-positive cells in SNc.

The observation that trkB receptors begin to be downregulated seven days after 6-OHDA injection is intriguing and raises the question whether this might be linked to transient increases of BDNF expression observed in SN at early times after neurotoxin injection [12, 13]. We have reported a substantial increased expression of BDNF transcripts in SN as early as 1–4 days after 6-OHDA intrastriatal injection, an effect that was abolished by MK-801, a nonselective antagonist of NMDA receptors, but not by Ifenprodil, selective antagonist of NR2B-containing NMDA receptors [11–13, 43]. As judged by ISH and IHC studies, such increases in BDNF expression appear to occur preferentially in SNR [12, 13]. In addition, such increases occur in parallel with mild but significant increases in extracellular levels of glutamate and aspartate in SN [11, 13]. At later stages (7 days) in this presymptomatic PD model, nigral BDNF transcripts expression started to return to basal levels, whereas glutamate and aspartate extracellular levels kept increasing quite dramatically in SN [13]. This last time point after striatal 6-OHDA injection seems to determine the initiation of trkB downregulation in SN as reported in this work. However and in contrast to BDNF upregulation, the changes in trkB expression shown occur predominantly in SNc and are selectively blocked by an antagonist of NR2B-containing NMDA receptor such as Ifenprodil. Therefore, such downregulation of trkB in SNc after neurotoxin injection could be triggered by an increased overflow of excitatory amino acids and a parallel activation of NR2B-NMDA receptors. Despite the previous considerations, it is not possible to disregard that increased endogenous BDNF levels in SN may contribute also to the neurotoxin-induced decrease of trkB observed in this midbrain subregion. Indeed, BDNR-IR cells in SNc and SNr remained significantly elevated at 7 days of this 6-OHDA presymptomatic model, although this coincided with BDNF transcripts expression actually returning to basal levels [13]. Therefore, elevated levels of BDNF protein in SNc might very well induce an internalization process and a later proteolysis of the trkB receptor. Supporting this idea, studies conducted in primary cultures of hippocampal neurons and cerebellar granule neurons demonstrate that exposure of these cultures to the BDNF ligand results in a decrease both in trkB mRNA and protein and that these changes are prevented by inhibitors of proteosomal function [44, 45]. It remains to be established whether such BDNF-dependent trkB internalization occurs in SNc at early times after 6-OHDA intrastriatal injections.

It is interesting that the 6-OHDA intrastriatal administration induces a downregulation of trkB receptor specifically in SNc while no effect is detected in SNR. According to the data presented in this work, trkB shows a lower expression in SNR than in SNc. As discussed before, early after neurotoxin injection an increased expression of BDNF transcripts occurs preferentially in SNR as compared to SNc [12, 13], while no change is detected in trkB expression in any of the SN subregions. Therefore, it would be possible to suggest that early after the 6-OHDA-induced injury there is an increased BDNF/trkB ratio in SNR compared to SNc, which might explain the differential effects induced by the toxin in each SN subregions. Additionally, relative differences in glutamate overflow and subsequent NR2B-NMDA receptors activation could also contribute to the differential trkB regulation shown here in these SN subregions. It is interesting that a subcellular relocation of NR2B-NMDA receptors from synaptic to extrasynaptic sites has been reported to occur in the striatum of rats after an acute injection of 6-OHDA in SN [46]. It remains to be determined whether such phenomenon also happens in SNc following intrastriatal 6-OHDA injection, our experimental condition. Another important consideration is associated with the fact that the decrease in trkB after 6-OHDA treatment occurs only in TH-IR cells located in SNc, positioning the DA cells as the main targets of this downregulation. Conversely, our results show that trkB coexists in SNR mainly with non-DAergic cells and in this SN subregion no change is observed. Therefore, a combination of DAegic cell phenotype, BDNF/trkB ratio, local glutamate overflow and relative predominance of NR2B-NMDA receptors could contribute to the early and specific downregulation of trkB receptors which is observed in SNc in this experimental model of presymptomatic PD.

### 4.3. NR2B-Containing NMDA Receptors Mediate the Decrease in TrkB in SNc

It has been shown that in both, the presymptomatic and symptomatic phases of PD, there is an increased glutamatergic drive over the SN arising mainly from the Subthalamic Nucleus (STN). This glutamatergic hyperactivity seems to be a response to the misregulation of output motor information from the basal ganglia, due to the progressive loss of dopaminergic inputs arriving to the striatum [32, 47]. In agreement with this proposition, we have previously shown an increase in extracellular glutamate levels in SN in this presymptomatic model of PD [13]. We have also provided evidence that supports the existence of a functional coupling between increased glutamatergic drive, NMDA receptor activation, and BDNF expression in SN in this rodent PD model [13].

In this regard, results presented in this work indicate that ifenprodil, a selective antagonist of NR2B-NMDA receptors, prevents the downregulation of trkB expression in SNc after striatal 6-OHDA injection. At the molecular level, it has been reported that one of the promoters described for trkB is repressed by increases in intracellular calcium levels [48], and therefore, it would be possible to suggest that an increased glutamatergic drive may induce the activation of calcium permeable NMDA receptors in SN, which could in turn mediate an increase in intracellular calcium levels finally leading to a reduction in trkB expression in this midbrain region.

Altogether, previous and current data allow us to suggest that glutamatergic drive and NMDA receptor activation may exert opposing effects on BDNF and trkB expression in SN during early PD. Essentially, they could be mediating a differential biphasic time pattern expression of the BDNF/trkB receptor complex, which precedes DA cell death in SN and the clinical symptoms of this neurological disease. Thus, early after striatal 6-OHDA injections (1 to 4 days in our rodent model of PD), increased synaptic glutamatergic information in SN would mediate the increase in BDNF expression, neurotrophin that may be associated with positive compensatory actions in SN. Later on (by 7 days), overactivity of glutamatergic pathways innervating SN would specifically activate NR2B-containing NMDA receptors causing a downregulation of trkB receptors. This would be consistent with the proposition that the NR2B-NMDA receptors are partly located extrasynaptically and that spillover from overactive synapses might be involved in the activation of these receptors [49–51]. Finally, a deficit in BDNF-mediated signaling may develop in SNc, which in turn might contribute to proapoptotic actions and to nigral dopaminergic neuronal death in this midbrain subregion. This is a proposition we are currently evaluating in our lab.

### 4.4. Is a Coupling between NR2B-NMDA Receptors and BDNF/TrkB Signaling Involved in the Maintenance of DA Cell Phenotype in SNc after 6-OHDA Intrastriatal Injections?

The study we describe here raises the question whether the NMDA receptor-mediated downregulation of trkB receptor in SN after 6-OHDA treatment occurs as part of compensatory events related to the survival and functional integrity of DA cells in SN. The changes reported here on trkB expression occurred mainly in SNc in a cellular localization coincident with that of TH, an important phenotypic marker of DA cells in SN. Therefore, DA cells were functionally deprived as early as 7 days after neurotoxin injection of an important neurotrophin receptor support via a mechanism involving NR2B-NMDA receptor activation. At a later time point (14 days) after 6-OHDA treatment, the number of Nissl-stained and TH-IR cells in SNc started to decrease through a mechanism which also involves NR2B-NMDA receptor, at least in the case of TH-IR. As shown here, most of the TH-IR cells coexist with trkB receptors in SNc, suggesting that an earlier disappearance of this receptor may render these cells more susceptible to 6-OHDA pro-oxidative effects. In view of the previous considerations, it seems no surprising that prior treatments with an NR2B-NMDA receptor antagonist such as Ifenprodil abolishes not only the reduction in the expression of trkB observed in SNc after 6-OHDA treatment but also the change in TH-IR. Both effects would occur most likely in DAergic cells located in this midbrain subregion. It should be mentioned that the existence of a mechanism which is NMDAR-dependent, but trkB-independent, could also account in part for the reduction of TH-IR in SNc after 6-OHDA treatment. However, by means of the present presymptomatic PD model, it was shown that systemic injection of K-252a, an inhibitor of trkB receptors, significantly anticipates the time point at which TH-IR cells start to disappear in rat SNc in response to 6-OHDA striatal injection [52]. In addition, recent observations in experimental models of PD in adult rodents indicate that compounds such as rasagiline and 7, 8-dihydroxyflavone, a trkB agonist, may induce DAergic neuronal protection in SN through trkB receptor activation [53, 54]. All these results support the suggestion that trkB receptors and NR2B-NMDA receptors may be important for the maintenance of the DAergic phenotype during presymptomatic stages of PD. Notwithstanding the previous considerations, further experiments on cell viability in SNc are necessary in this PD model with specific markers of DAergic cells other than TH to strength the proposition that NMDA receptors and BDNF/trkB signaling are necessary to maintain DAergic neuronal survival. In this regard, consistent with this idea it was shown that old mutant mice with haploinsufficiency for trkB showed a greater loss of DAergic neurons when compared to old wild-type animals [17].

## 5. Conclusions

These results and those previously reported by us [13], conducted in a rat model of PD, suggest that both BDNF and trkB expression and function may be tightly regulated in SN during the presymptomatic stages of PD. Moreover, the results presented here give further support to the idea that a functional coupling between NMDA receptors and BDNF/trkB signaling may be important for the maintenance of the dopaminergic phenotype in SNc during the presymptomatic stages of this neurological disease.
